# Supplementary material for: Rbm10 facilitates heterochromatin assembly via the Clr6 HDAC complex
Source: Epigenetics Chromatin. 2021 Jan 19;14:8. doi: 10.1186/s13072-021-00382-y (PMC7816512; doi:10.1186/s13072-021-00382-y)
Supplement: Supplementary file 1 — Additional file 1: Figure S1. Sequence alignment of fission yeast Rbm10 (YDMD_SCHPO) with Rbm10 orthologs in Callithrix jacchus, human, Macaca mulatta, mouse and rat. [file 13072_2021_382_MOESM1_ESM.docx]

**Rbm10 facilitates heterochromatin assembly via the Clr6 HDAC complex**

Martina Weigt, Qingsong Gao, Hyoju Ban, Haijin He, Guido Mastrobuoni and Stefan Kempa, Wei Chen, and Fei Li

**
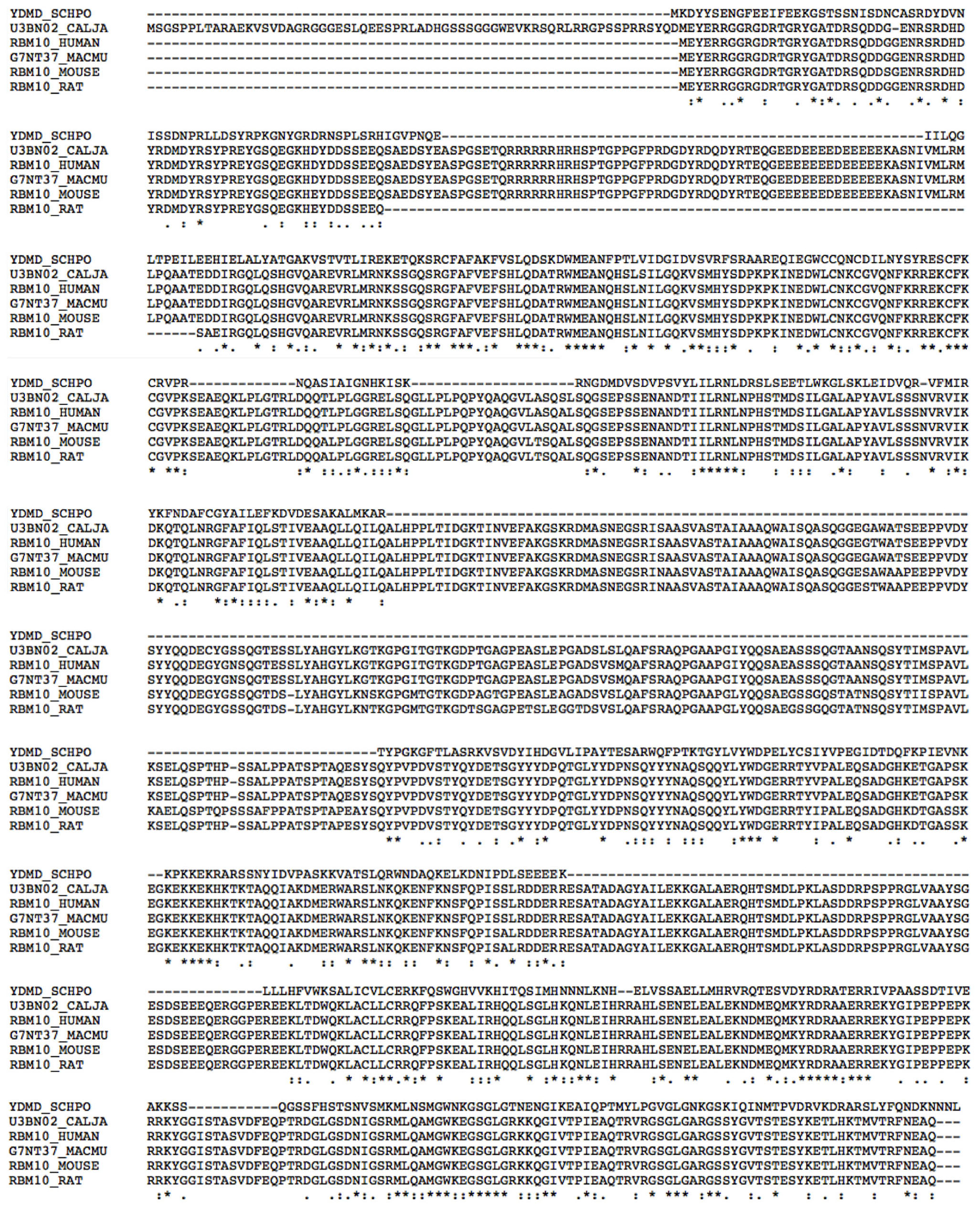
**

**Figure S1. Sequence alignment of fission yeast Rbm10 (YDMD_SCHPO) with Rbm10 orthologs in *Callithrix jacchus*, human, *Macaca mulatta,* mouse and rat.**
