# Supplementary material for: Rbm10 facilitates heterochromatin assembly via the Clr6 HDAC complex
Source: Epigenetics Chromatin. 2021 Jan 19;14:8. doi: 10.1186/s13072-021-00382-y (PMC7816512; doi:10.1186/s13072-021-00382-y)
Supplement: Supplementary file 9 — Additional file 9: Figure S2. Western blot analysis of indicated cells expressing Alp13-GFP using an anti-GFP antibody. Ponceau staining was used as a loading control. [file 13072_2021_382_MOESM9_ESM.docx]

**Rbm10 facilitates heterochromatin assembly via the Clr6 HDAC complex**

Martina Weigt, Qingsong Gao, Hyoju Ban, Haijin He, Guido Mastrobuoni and Stefan Kempa, Wei Chen, and Fei Li


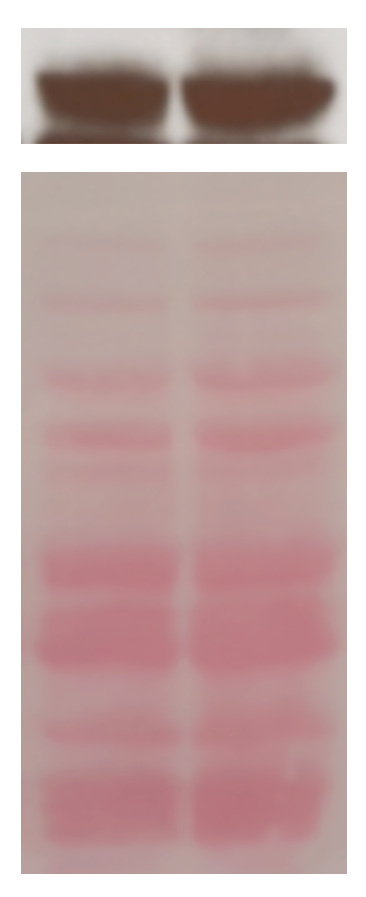


*rbm10*Δ

WT

Alp13-GFP

Ponceau

**Figure S2.** Western blot analysis of indicated cells expressing Alp13-GFP using an anti-GFP antibody. Ponceau staining was used as a loading control.
