# Supplementary material for: Rbm10 facilitates heterochromatin assembly via the Clr6 HDAC complex
Source: Epigenetics Chromatin. 2021 Jan 19;14:8. doi: 10.1186/s13072-021-00382-y (PMC7816512; doi:10.1186/s13072-021-00382-y)
Supplement: Supplementary file 10 — Additional file 10: Figure S3. Analysis of the association of Alp13-GFP with the rDNA region in the indicated strains by ChIP. ChIP assays were performed using an antibody against GFP. act1+ was used as a control. [file 13072_2021_382_MOESM10_ESM.docx]

**Rbm10 facilitates heterochromatin assembly via the Clr6 HDAC complex**

Martina Weigt, Qingsong Gao, Hyoju Ban, Haijin He, Guido Mastrobuoni and Stefan Kempa, Wei Chen, and Fei Li


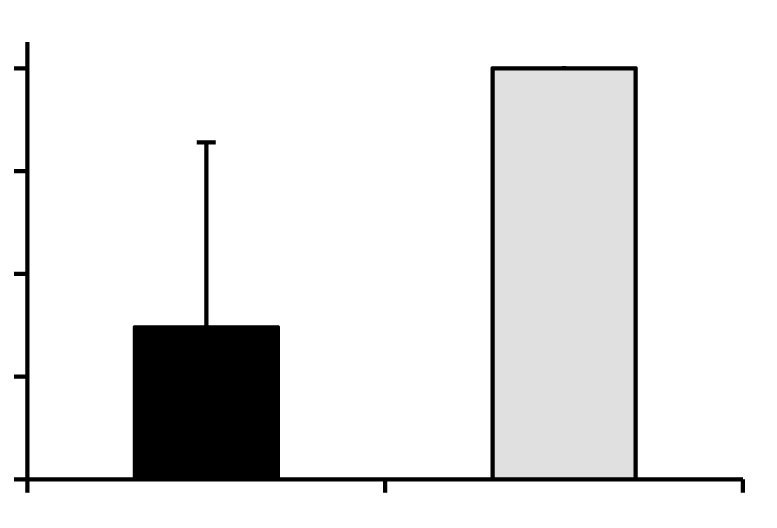


Alp13-GFP enrichment

at rDNA

100

50

150

200

WT

*rbm10*Δ

**Figure S3. Analysis of the association of Alp13-GFP with the rDNA region in the indicated strains by ChIP.** ChIP assays were performed using an antibody against GFP. *act1^+^* was used as a control.
