# Supplementary material for: Rbm10 facilitates heterochromatin assembly via the Clr6 HDAC complex
Source: Epigenetics Chromatin. 2021 Jan 19;14:8. doi: 10.1186/s13072-021-00382-y (PMC7816512; doi:10.1186/s13072-021-00382-y)
Supplement: Supplementary file 11 — Additional file 11: Table S8. Strains used in this study. [file 13072_2021_382_MOESM11_ESM.docx]

**Additional Table S8. Strains used in this study**

| **Strain** | **Genotype** | **Figures** |
| --- | --- | --- |
| FL7 | *h^90^ otr1RSph1::ade6 TEL 1L-his3 ade6-210 his3-D1 leu1-32 ura4-DS/E* | Fig. 4B |
| FL24 | *h^+^ otr1R(SphI)::ura4+ leu1-32 ade6-210 ura4-DS/E his1-10* | Fig. 4A |
| FL97 | *h^90^ mat3-M::ura4 ade6-210 leu1-32 ura4-DS/E* | Fig. 4C |
| FL455 | *h^-^ rbm10Δ::KanMX6 ura4-D18 leu1-32 ade6-210 his3-D1* | Fig. 3, 4D |
| FL479 | *h^-^ rbm10-GFP-KanMX6 ura4-D18 leu1-32 ade6-210 his3-D1* | Fig. 1B |
| FL529 | *h^-^  pREP1-FLAG-HA-rbm10 ura4-D18 ade6-210 his3-D1 leu1-32* | Fig. 2, 5B-D |
| MG01 | *h^-^  FLAG-HA-rbm10-KanMX ura4-D18 ade6-210 his3-D1 leu1-32* | Fig. 5A |
| MG02 | *h^-^ rbm10Δ::KanMX6 otr1R(SphI)::ura4+ leu1-32 ade6-210 ura4-D18 his1-10* | Fig. 4A |
| MG03 | *h^-^ rbm10Δ::KanMX6 TEL 1L-his3 ade6-210 his3-D1 leu1-32 ura4-DS/E* | Fig. 4B |
| MG04 | *h^-^ rbm10Δ::KanMX6 mat3-M::ura4 ade6-210 leu1-32 ura4-DS/E* | Fig. 4C |
| FL780 | *h^90^ alp13::alp13-GFP-HA-KanMX6 ade6-216 leu1-32 lys1-131 ura4-D18* | Fig. 6B |
| FL781 | *h^?^ alp13::alp13-GFP-HA-KanMX6 rbm10-TAP-KanMX6 ade6-216 leu1-32 lys1-131 ura4-D18* | Fig. 6B |
| FL782 | *h^?^ alp13::alp13-GFP-HA-KanMX6 rbm10Δ::NatR ade6-216 leu1-32 lys1-131 ura4-D18* | Fig. 6C |
